# Synthesis and Biological Activity of *trans*-Tiliroside Derivatives as Potent Anti-Diabetic Agents

**DOI:** 10.3390/molecules15129174

**Published:** 2010-12-10

**Authors:** Yujin Zhu, Yanjun Zhang, Yi Liu, Hongwan Chu, Hongquan Duan

**Affiliations:** 1 School of Pharmaceutical Sciences, Research Center of Basic Medical Sciences, Tianjin Medical University, Tianjin 300070, China; E-Mails: wjyxyzyj@yahoo.com.cn (Y.J.Z.); aliu_yi@yahoo.com.cn (Y.L.); chw40542135@yahoo.com.cn (H.W.C.); 2 Chinese People’s Armed Police Forces, Tianjin Provincial Corps Hospital, Tianjin 300162, China; 3 Tianjin University of Traditional Chinese Medicine, Tianjin 300193, China; E-Mail: zyjsunye@163.com (Y.J.Z.)

**Keywords:** *trans***-**tiliroside, derivative synthesis, Novozyme435, anti-diabetic activity

## Abstract

A set of novel *trans*-tiliroside derivatives were synthesized. The structures of the derivatives were identified by their IR, ^1^H-NMR, and MS spectra analysis. Their anti-diabetic activities were evaluated on the insulin resistant (IR) HepG2 cell model. As a result, compounds **7a**, **7c**, **7h**, and *trans-*tiliroside exhibited significant glucose consumption-enhancing effects in IR-HepG2 cells compared with the positive control (metformin). This research provides useful clues for further design and discovery of anti-diabetic agents.

## 1. Introduction

The complex metabolic syndrome, diabetes mellitus, is a major worldwide human health concern that is estimated to affect 300 million people by the year 2025 [1]. Most of the patients who have diabetes have non-insulin dependent diabetes mellitus (NIDDM). Resistance to the biological actions of insulin in the liver and peripheral tissues, together with pancreatic cell defects, is a major feature of the pathophysiology of human NIDDM [2,3,4]. 

**Figure 1:**
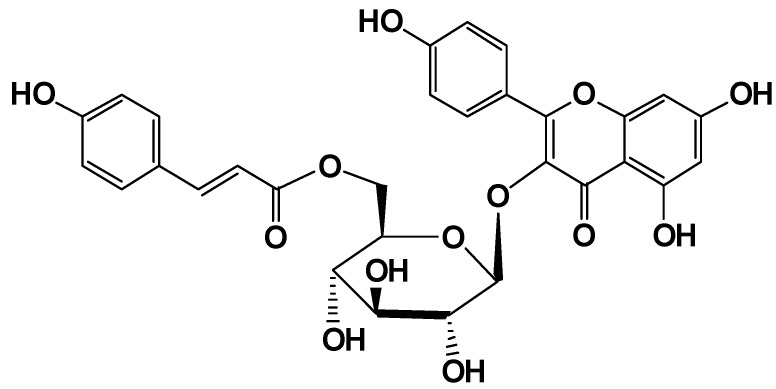
The structure of *trans*-tiliroside.

In a previous study of traditional Chinese herbal medicines, we were particularly interested in the extraction and separation of anti-hyperglycemic constituents from *Potentilla chinensis* Ser. (Rosaceae) which has been reported having anti-hyperglycemic activity in the clinic [5]. Further photochemical and bioactive analysis studies afforded an anti-diabetic compound, kaempferol-3-*O*-β-D-(6-*O*-*trans*- *p*-cinnamoyl)glucopyranoside (*trans*-tiliroside, Figure 1), which revealed significant anti-hyperglycemic effects when compared with phenethyldiguanide in alloxan mice [6]. As a part of *trans*-tiliroside, kaempferol-3-*O*-β-D-glucopyranose (**4**) and related analogues revealed weak anti-diabetes activity [7,8], so the cinnamoyl part of *trans*-tiliroside is presumed to be the the critical factor for increasing the activity. This paper deals with the synthesis of a series of novel *trans*-tiliroside derivatives by replacing the cinnamoyl ring, as well as their biological activities.

## 2. Results and Discussion

### 2.1. Synthesis

**Scheme 1:**
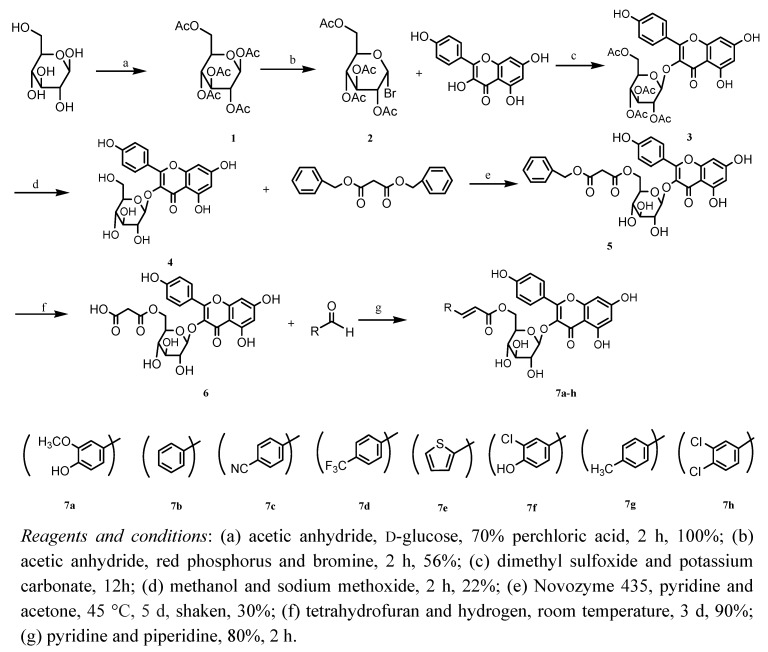
Synthesis of compounds 7a-7h.

In the current work, a set of novel *trans*-tiliroside derivatives were synthesized, among which compounds **5**, and **7c-7h** have not been reported before. All relevant reactions are depicted in Scheme 1. 6-(Acetoxymethyl)tetrahydro-2*H*-pyran-2,3,4,5-tetrayl tetraacetate (**1**) was prepared by treating anhydrous D-glucose with perchloric acid in acetic anhydride (yield 100%). Treatment of **1** with bromine and red phosphorus produced **2**, which was treated with kaempferol in DMSO in the presence of excess anhydrous potassium carbonate at room temperature to yield compound **3**. Compound **4** was prepared by treating **3** with sodium methoxide in methanol. Shaking compound **4 **with dibenzyl malonate at 45 °C for 120 hours led to **5** in 30% yield. Catalytic hydrogenation of **5 **on Pd/C in THF solution afforded pure **6 **in quantitative yield after catalyst filtration and solvent evaporation at room temperature. Compound **6** was used as a key intermediate for the synthesis of derivatives **7a-7h**, which were obtained by the Knoevenagel-Doebner reaction of **6** with a series of aldehydes in the presence of pyridine [9,10,11,12,13,14,15]. The derivatives were characterized by IR, ^1^H-NMR, and MS spectroscopy.

### 2.2. Biological Activity

In this research, an insulin resistant (IR) HepG2 cell model [16,17,18] was used for appraising the anti-hyperglycemic effectws of the synthesized *trans-*tiliroside derivatives. As a result, compounds **7a**, **7c**, **7h**, and *trans-*tiliroside itself all exhibited significant glucose consumption-enhancing effects in IR-HepG2 cells compared with a positive control (metformin; Table 1). From the *in vitro* EC_50_ values we presume that *meta* and *para*-substitution in the benzene ring of cinnamoyl with electron withdrawing groups such as cyano-group and chloride group in compounds **7c** and **7h** resulted in enhanced anti-diabetic activity. 

So far, many anti-diabetic flavonoids have been reported [19,20,21,22], such as myricetin with insulinomimetic effects [23], quercetin with antidiabetic effects in STZ diabetic rats [24], and kaempferol-3,7-*O*-(α)-dirhamnoside with hypoglycemic and antioxidant effects [7,8]. However, the abovementioned flavonoids had weak activity as revealed by their high doses and comparison with market drugs. *trans*-Tiliroside is a known natural compound, and its anti-oxidant [25], tyrosinase inhibitory [26], anti-complement activity [27], acetylcholinesterase inhibition [28], and anti-obesity effects [29] have been reported. For the first time, we synthesized a series of trans-tiliroside derivatives, which revealed significant anti-diabetic activities compared with the market drug of Metformin. The results suggest that trans-tiliroside derivatives can be considered promising candidates in the development of new antidiabetic lead compound. Further biological evaluations are in progress.

**Table 1:** Effects on glucose consumption of tiliroside derivatives in IR HepG2 cells.

Compound	EC_50_ (µM)
**7a**	0.109
**7b**	>10
**7c**	0.048
**7d**	>10
**7e**	>10
**7f**	>10
**7g**	0.813
**7h**	0.011
*trans*-tiliroside	0.155
metformin	0.270

## 3. Experimental

### 3.1. General

All reactions were monitored by TLC on silica gel GF-254 plates purchased from Qingdao Haiyang Corporation.^ 1^H-NMR and ^13^C-NMR spectra were taken on a Bruker AV400 MHz. DMSO-d_6_ or CD_3_OD were used as the solvent. Chemical shifts are reported in parts per million shift (δ value) from Me_4_Si (δ 0 ppm for ^1^H). Melting points were measured with an X4 melting apparatus. IR spectra were recorded on a Nicolet 380 spectrometer (KBr). HRMS was taken on a Varian 7.0T HRFTICR-MS.

### 3.2. Materials

*2-(Acetoxymethyl)-6-bromotetrahydro-2H-pyran-3,4,5-triyl-triacetate* (**2**). To a mixture of acetic anhydride (100 mL) and D-glucose (0.5 g, 2.78 mmol), 70% perchloric acid (0.6 mL) was added dropwise. The mixture was then stirred at 35-40 °C on a water bath until the solution became clear. After that, anhydrous D-glucose (24.5 g, 0.136 mol) was added over a period of 0.5 h and the reaction continued for 2 h. The mixture was then cooled to 20-25 °C. Red phosphorus (7.5 g) and bromine (14.5 mL) was added to the reaction mixture. After 0.5 h, H_2_O (9 mL) was added and the reaction continued for 2 h. When the reaction ended, chloroform (75 mL) was added to the mixture and the resulting reaction mixture was filtered. The filtrate was then poured into ice water, extracted with chloroform (40 mL), washed with cold water and saturated sodium carbonate, dried with anhydrous magnesium sulfate, filtered and evaporated. The resulting solid was finally re-crystallized with ether on an ice bath and afforded product as a white solid (31 g, 56% yield). m.p. 88-89 °C.

*5,7-Dihydroxy-2-(4-hydroxyphenyl)-3-(3,4,5-trihydroxy-6-(hydroxymethyl)tetrahydro-2H-pyran-2-yl-oxy)-4H-chromen-4-one* (**4**). Kaempferol (650 mg, 2.27 mmol) and tetraacetyl-α-1-bromoglucose (2.7 g, 6.55 mmol) were dissolved in dimethyl sulfoxide (50 mL) and then stirred overnight in the presence of potassium carbonate. The resulting mixture was then adjusted to an acidic pH by adding a few drops of formic acid. The precipitate formed in the acidic solution was separated by centrifugation, washed and concentrated. Anhydrous MeOH (100 mL) was added to the precipitate, the solution was adjusted to pH = 8 with Sodium methoxide and kept at room temperature for 2 h, followed by neutralization and filtration. The filtrate was subjected to silica gel FC (AcOEt/MeOH/AcOH, 5:1:0.5). The product was obtained as a yellow solid (240 mg, 22% yield). m.p. 163-164 °C. ^1^H-NMR (400 MHz, DMSO), δ: 8.04 (2H, *d*, *J* = 8.8 Hz, 2′,6′-H), 6.88 (2H, *d*, *J* = 8.8 Hz, 3′,5′-H), 6.44 (1H, *br s*, 8-H), 6.21 (1H, *br s*, 6-H), 5.45 (1H, *d*, *J* = 7.4 Hz, Gal H-1), 4.01-4.21 (2H, *m*, 6′′-H), 3.50-3.20 (4H, *m*, 2′′, 3′′, 4′′, 5′′-H). ESI-MS: *m/z* [M+Na]^+^, calcd. For C_21_H_20_O_11_Na: 471.1; found: 471.1.

*Benzyl(6-(5,7-dihydroxy-2-(4-hydroxyphenyl)-4-oxo-4a,8a-dihydro-4H-chromen-3-yloxy)-3,4,5-tri-hydroxytetrahydro-2H-pyran-2-yl)methyl malonate* (**5**). To a solution of the flavonol glycoside **4 **(125 mg, 0.28 mmol) in anhydrous Me_2_CO were added 10% pyridine (3 mL), dibenzyl malonate (15 equiv) and Novozyme 435 (600 mg) were added, and the suspension was shaken at 45 °C and 250 rpm for 5 days. After filtration of the enzyme and evaporation of the solvent, the residue was repeatedly washed with hexane five times and then purified by column chromatography using AcOEt/MeOH (3:1) as eluent. The product was obtained as a yellow solid (50 mg, 30% yield). m.p. 214-215 °C. ^1^H-NMR (400 MHz, CD_3_OD), δ: 7.95 (2H, *d*, *J* = 9.0 Hz, 2′,6′-H), 7.21-7.31 (5H, *m*, -ph), 6.80 (2H, *d*, *J* = 9.0 Hz, 3′,5′-H), 6.30 (1H, *d*, *J* = 1.8 Hz, 8-H), 6.13 (1H, *d*, *J* = 1.8 Hz, 6-H), 5.12 (1H, *d*, *J* = 7.2 Hz, 1′′-H), 4.96-5.12 (2H, *m*, ph-CH_2_-O), 4.05-4.24 (2H, *m*, 6′′-H), 3.39 (3H, *m*, 2′′,3′′,4′′-H), 3.27 (1H, *m*, 5′′-H), 3.27 (2H, s, -CO-CH_2_-CO-). ESI-MS: *m/z* [M-H]^-^ , calcd. For C_31_H_27_O_14_: 623.1; found: 623.1.

*3-((6-(5,7-Dihydroxy-2-(4-hydroxyphenyl)-4-oxo-4H-chromen-3-yloxy)-3,4,5-trihydroxytetrahydro-2H-pyran-2-yl)methoxy)-3-oxopropanoic acid* (**6**). A solution of the 3-flavone glycoside benzyl malonate **5 **(200 mg, 0.32 mmol) in anhydrous THF (5 mL) was stirred with a catalytic amount of Pd/C (5%) for 3 days under a H_2_ atmosphere. The catalyst was filtered and solvent was removed under vacuum at room temperature to afford the malonyl glycoside in quantitative yield as a yellow solid (150 mg, 90% yield). m.p. 178-179 °C. ^1^H-NMR (400 MHz, CD_3_OD), δ: 7.96 (2H, *d*, *J* = 8.0 Hz, 2′,6′-H), 6.87 (2H, *d*, *J* = 8.0 Hz, 3′,5′-H), 6.43 (1H, *br s*, 8-H), 6.20 (1H, *br s*, 6-H), 5.34 (1H, *d*, *J* = 6.8 Hz, 1′′-H), 3.99-4.18 (2H, *m*, 6′′-H), 3.23 (3H, *m*, 2′′,3′′,4′′-H), 3.20 (1H, *m*, 5′′-H), 3.07 (2H, *s*, -CO-CH_2_-CO-). ESI-MS: *m/z* [M-H]^-^ , calcd. For C_24_H_21_O_14_: 533.1; found: 533.1. 

*(E)-(6-(5,7-Dihydroxy-2-(4-hydroxyphenyl)-4-oxo-4H-chromen-3-yloxy)-3,4,5-trihydroxytetrahydro-2H-pyran-2-yl)methyl3-(4-hydroxy-3-methoxyphenyl)acrylate* (**7a**). Crude **6** (24.6 mg, 0.046 mmol) was dissolved in anhydrous pyridine (3 mL) containing 4-hydroxy-3-methoxybenzaldehyde (60 µL, 3 equiv.) and piperidine (20 µL). After the addition of molecular sieves, the mixture was heated at 60 °C for 2.5 h. Usual workup and purification by FC (AcOEt/MeOH/AcOH, 10:1:0.5) gave **7a** as a yellow power (23 mg, 80% yield). m.p. 203-204 °C. IR(KBr), ν_max_ cm^-1 ^3392, 1649, 1597, 1512. ^1^H-NMR (400 MHz , DMSO-d_6_), δ: 7.99 (2H, *d*, *J* = 8.8 Hz, 2′,6′-H), 7.39 (1H, *d*, *J* = 15.8 Hz, 3′′′-H), 7.22 (1H, *s*, 5′′′-H), 6.94 (1H, *d*, *J* = 7.8 Hz, 9′′′-H), 6.80 (1H, *d*, *J* = 7.8 Hz, 8′′′-H), 6.85 (2H, *d*, *J* = 8.8 Hz, 3′,5′-H), 6.27 (1H, *br s*, 8-H), 6.06 (1H, *br s*, 6-H), 6.28 (1H, *d*, *J* = 15.8 Hz, 2′′′-H), 5.42 (1H, *d*, *J* = 7.2 Hz, 1′′-H), 4.21-4.31 (2H, *m*, 6′′-H), 3.47 (3H, *m*, 2′′, 3′′, 4′′-H), 3.34 (1H, *m*, 5′′-H). HR ESI-MS: *m/z* [M+Na]^+^ , calcd. For C_31_H_28_O_14_Na: 647.1377; found: 647.1362. Compounds **7b**-**7h** were synthesized in the same manner.

*(E)-(6-(5,7-Dihydroxy-2-(4-hydroxyphenyl)-4-oxo-4H-chromen-3-yloxy)-3,4,5-tri-hydroxytetrahydro- H-pyran-2-yl)methyl cinnamate* (**7b**). Evaporation of the solvent gave **7b** as a yellow power (79%); m.p. 231-232 °C. IR (KBr) ν_max_ cm^-1^: 3374, 1654, 1606, 1503. ^1^H-NMR (400 MHz, CD_3_OD), δ: 8.00 (2H, *d*, *J* = 8.8 Hz, 2′,6′-H), 7.38-7.48 (5H, *m*, 5′′′~9′′′-H), 7.42 (1H, *d*, *J* = 16.0 Hz, 3′′′-H), 6.82 (2H, *d*, *J* = 8.8 Hz, 3′,5′-H), 6.29 (1H, *d*, *J* = 2.0 Hz, 8-H), 6.25 (1H, *d*, *J* = 16.0 Hz, 2′′′-H), 6.11 (1H, *d*, *J* = 2.0 Hz, 6-H), 5.25 (1H, *d*, *J* = 7.2 Hz, 1′′-H), 4.21-4.30 (2H, *m*, 6′′-H), 3.45 (3H, *m*, 2′′,3′′,4′′-H), 3.35 (1H, *m*, 5′′-H). HR ESI-MS: *m/z* [M+Na]^+^ , calcd. For C_30_H_26_O_12_Na: 601.1322; found: 601.1319.

*(E)-(6-(5,7-Dihydroxy-2-(4-hydroxyphenyl)-4-oxo-4H-chromen-3-yloxy)-3,4,5-trihydroxytetrahydro-2H-pyran-2-yl)methyl 3-(4-cyanophenyl)acrylate* (**7c**). Evaporation of the solvent gave **7c** as a yellow power (75%); m.p. 217-218 °C. IR (KBr), ν_max_cm^-1^ 3407, 1643, 1601, 1507. ^1^H-NMR (400 MHz, DMSO-d_6_), δ: 7.96 (2H, *d*, *J* = 8.2 Hz, 2′,6′-H), 7.84 (2H, *d*, *J* = 8.0 Hz, 5′′′, 9′′′-H), 7.71 (2H, *d*, *J* = 8.0 Hz, 6′′′, 8′′′-H), 7.44 (1H, *d*, *J* = 16.0 Hz, 3′′′-H), 6.84 (2H, *d*, *J* = 8.2 Hz, 3′,5′-H), 6.51 (1H, *d*, *J* = 16.0 Hz, 2′′′-H), 6.23 (1H, *br s*, 8-H), 5.98 (1H, *br s*, 6-H), 5.44 (1H, *d*, *J* = 7.5 Hz, 1′′-H), 4.21-4.30 (2H, *m*, 6′′-H), 3.47 (3H, *m*, 2′′, 3′′, 4′′-H), 3.35 (1H, *m*, 5′′-H). HR ESI-MS: *m/z* [M+Na]^+ ^, calcd. For C_31_H_25_NO_12_Na: 626.1274; found: 626.1257. 

*(E)-(6-(5,7-Dihydroxy-2-(4-hydroxyphenyl)-4-oxo-4H-chromen-3-yloxy)-3,4,5-trihydroxytetrahydro-2H-pyran-2-yl)methyl3-(4-(trifl uoromethyl) phenyl) acrylate* (**7d**). Evaporation of the solvent gave **7d** as a yellow power (81%); m.p. 189-190 °C. IR (KBr), ν_max _cm^-1 ^3412, 1647, 1611, 1509. ^1^H-NMR (400 MHz, DMSO-d_6_), δ: 7.98 (2H, *d*, *J* = 8.8 Hz, 2′, 6′-H), 7.74 (4H, 6′′′, 8′′′, 5′′′, 9′′′-H), 7.49 (1H, *d*, *J* = 16.0 Hz, 3′′′-H), 6.85 (2H, *d*, *J* = 8.8 Hz, 3′, 5′-H), 6.50 (1H, *d*, *J* = 16.0 Hz, 2′′′-H), 6.29 (1H, *br s*, 8-H), 6.04 (1H, *br s*, 6-H), 5.44 (1H, *d*, *J* = 7.5 Hz, 1′′-H), 4.13-4.31 (2H, *m*, 6′′-H), 3.34 (3H, *m*, 2′′, 3′′, 4′′-H), 3.25 (1H, *m*, 5′′-H). HR ESI-MS: *m/z* [M+Na]^+^ , calcd. For C_31_H_25_F_3_O_12_Na: 669.1196; found: 669.1189.

*(E)-((3S,4R,6R)-6-(5,7-Dihydroxy-2-(4-hydroxyphenyl)-4-oxo-4H-chromen-3-yloxy)-3,4,5-trihydroxytetrahydro-2H-pyran-2-yl)methyl 3-(thiophen-2-yl) acrylate* (**7e**). Evaporation of the solvent gave **7e** as a yellow power (72%); m.p. 170-171 °C. IR (KBr), ν_max _cm^-1^ 3375, 1654, 1609, 1505. ^1^H-NMR (400 MHz, DMSO-d_6_), δ: 7.97 (2H, *d*, *J* = 9.0 Hz, 2′, 6′-H), 7.71 (1H, *d*, *J* = 5.4 Hz, 8′′′-H), 7.58 (1H, *d*, *J* = 15.6 Hz, 3′′′-H), 7.40 (1H, *d*, *J* = 3.6 Hz, 6′′′-H), 7.13 (1H, *dd*, *J* = 4.8 Hz, 7′′′-H), 6.84 (2H, *d*, *J* = 9.0 Hz, 3′, 5′-H), 6.31 (1H, *d*, *J* = 1.8 Hz, 8-H), 6.09 (1H, *d*, *J* = 1.8 Hz, 6-H), 6.01 (1H, *d*, *J* = 15.6 Hz, 2′′′-H), 5.40 (1H, *d*, *J* = 7.2 Hz, 1′′-H), 4.08-4.26 (2H, *m*, 6′′-H), 3.23 (3H, *m*, 2′′, 3′′, 4′′-H), 3.18 (1H, *m*, 5′′-H). HR ESI-MS: *m/z* [M+Na]^+^ , calcd. For C_28_H_24_SO_12_Na: 607.0886; found:607.0879.

*(E)-(6-(5,7-Dihydroxy-2-(4-hydroxyphenyl)-4-oxo-4H-chromen-3-yloxy)-3,4,5-trihydroxytetrahydro-2H-pyran-2-yl)methyl3-(3-chlo ro -4-hydroxyphenyl) acrylate* (**7f**). Evaporation of the solvent gave **7f** as a yellow power (76%); m.p. 200-201 °C. IR (KBr), ν_max _cm^-1 ^3415, 1650, 1601,1505. ^1^H-NMR (400 MHz, DMSO-d_6_), δ: 7.98 (2H, *d*, *J* = 8.3 Hz, 2′,6′-H), 7.57 (1H, *br s*, 5′′′-H), 7.34 (1H, *d*, *J* = 15.8 Hz, 3′′′-H), 7.28 (1H, *br d*, *J* = 8.4 Hz, 9′′′-H), 6.90 (1H, *d*, *J* = 8.4 Hz, 8′′′-H), 6.84 (2H, *d*, *J* = 8.3 Hz, 3′,5′-H), 6.37 (1H, *br s*, 8-H), 6.16 (1H, *d*, *J* = 15.8 Hz, 2′′′-H), 6.09 (1H, *br s*, 6-H), 5.41 (1H, *d*, *J* = 8.7 Hz, 1′′-H), 4.21-4.30 (2H, *m*, 6′′-H), 3.45 (3H, *m*, 2′′,3′′,4′′-H), 3.35 (1H, *m*, 5′′-H). HR ESI-MS: *m/z* [M+Na]^+ ^, calcd. For C_30_H_25_Cl O_13_Na: 651.0881; found: 651.0880.

*(E)-(6-(5,7-Dihydroxy-2-(4-hydroxyphenyl)-4-oxo-4H-chromen-3-yloxy)-3,4,5-trihydroxytetrahydro-2H-pyran-2-yl)methyl 3-p-tolylacrylate* (**7g**). Evaporation of the solvent gave **7g** as a yellow power(83%); m.p. 219-220 °C. IR (KBr), ν_max _cm^-1^ 3419, 1650, 1605, 1509. ^1^H-NMR (400 MHz, CD_3_OD), δ: 7.99 (2H, *d*, *J* = 8.3 Hz, 2′, 6′-H), 7.44 (1H, *d*, *J* = 16.2 Hz, 3′′′-H), 7.34 (2H, *d*, *J* = 7.8 Hz, 5′′′, 9′′′-H), 7.21 (2H, *d*, *J* = 7.8 Hz, 6′′′, 8′′′-H), 6.82 (2H, *d*, *J* = 8.3 Hz, 3′, 5′-H), 6.26 (1H, *br s*, 8-H), 6.21 (1H, *d*, *J* = 16.2 Hz, 2′′′-H), 6.12 (1H, *br s*, 6-H), 5.20 (1H, *d*, *J* = 7.2 Hz, 1′′-H), 4.21-4.30 (2H, *m*, 6′′-H), 3.50 (3H, *m*, 2′′, 3′′, 4′′-H), 3.36 (1H, *m*, 5′′-H), 2.38 (3H, *s*, -CH_3_). HR ESI-MS: *m/z* [M+Na]^+^ , calcd. For C_31_H_28_O_12_Na: 615.1478; found: 615.1467. 

*(E)-(6-(5,7-Dihydroxy-2-(4-hydroxyphenyl)-4-oxo-4H-chromen-3-yloxy)-3,4,5-trihydroxytetrahydro-2H-pyran-2-yl)methyl 3-(3,4-dichlorophenyl)acrylate* (**7h**). Evaporation of the solvent gave **7h** as a yellow power (70%);m.p. 183-184 °C. IR (KBr), ν_max_ cm^-1^ 3387, 1646, 1606, 1509. ^1^H-NMR (400 MHz, DMSO-d_6_), δ: 7.95 (2H, *d*, *J* = 9.0 Hz, 2′, 6′-H), 7.68 (1H, *s*, 5′′′-H), 7.66 (1H, *d*, *J* = 15.6 Hz, 3′′′-H), 7.61 (1H, *d*, *J* = 8.4 Hz, 9′′′-H), 7.42 (1H, *d*, *J* = 8.4 Hz, 8′′′-H), 6.83 (2H, *d*, *J* = 9.0 Hz, 3′, 5′-H), 6.36 (1H, *br s*, 8-H), 6.32 (1H, *d*, *J* = 15.6 Hz, 2′′′-H), 6.16 (1H, *br s*, 6-H), 5.41 (1H, *d*, *J* = 7.2 Hz, 1′′-H), 4.09-4.28 (2H, *m*, 6′′-H), 3.36 (3H, *m*, 2′′, 3′′, 4′′-H), 3.21 (1H, *m*, 5′′-H). HR ESI-MS: *m/z* [M+Na]^+^ , calcd. For C_30_H_24_Cl_2_O_12_Na: 669.0543; found: 669.0542.

### 3.3. Biological Activity

Cell culture and insulin resistant HepG2 model: HepG2 cells were cultured in high-glucose DMEM supplemented with 10% FBS. After confluence, cells were cultured in 96-well cluster plates in high-glucose DMEM supplemented with 10% FBS for 24 h, and then the cells were treated with 10^-7 ^mol/L insulin for 36 h in serum-free and phenol red-free high-glucose DMEM. After 36 h high concentration insulin stimulated, the cells were washed with pH = 4 high-glucose DMEM four times and PBS two times, then serum-free and phenol red-free high-glucose DMEM was added in with compounds in different concentrations and incubated for 24 h. After 24 h, the glucose content in the culture medium was measured by a glucose assay kit to study the effect on glucose consumption of insulin resistance HepG2. The enhancement ratio of glucose consumption (GC) was calculated as follows: GC % = (drug group of GC – model group of GC) / model group of GC × 100. 

## 4. Conclusions

In this research, eight novel *trans*-tiliroside derivatives were synthesized and characterized by IR, ^1^H-NMR, and MS analyses. Preliminary bioassay data indicates that the target compounds **7a**, **7c**, **7h**, and *trans*-tiliroside showed anti-hyperglycemic activities compared to the reference compound metformin. Further biological evaluations and the mechanism of the active compounds are in progress.
